# Tuberculoma of Eloquent Brain

**DOI:** 10.4269/ajtmh.21-0290

**Published:** 2021-05-10

**Authors:** Madhivanan Karthigeyan, Pravin Salunke, Anshul Siroliya

**Affiliations:** Department of Neurosurgery, Postgraduate Institute of Medical Education & Research, Chandigarh, India

**Figure 1.**
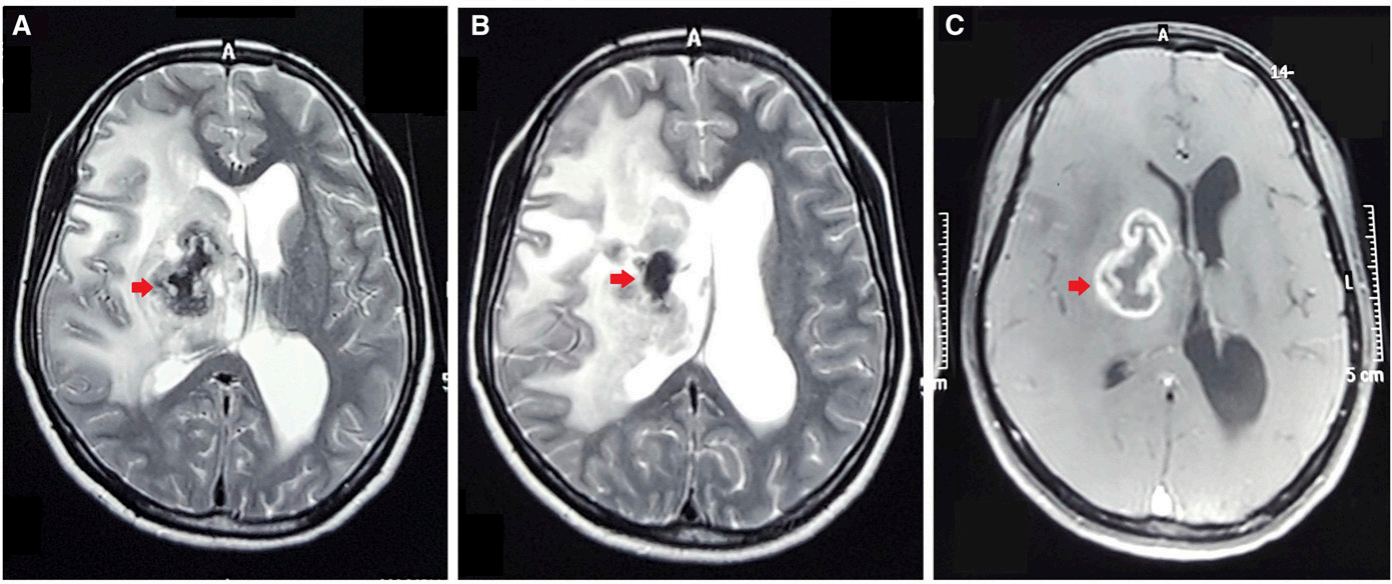
(**A**, **B**) Tuberculoma in the right basal ganglia with markedly hypointense center (arrow) in T2-weighted magnetic resonance imaging and (**C**) ring enhancement on contrast administration. This figure appears in color at www.ajtmh.org.

A 26-year-old woman with a low socioeconomic background living in rural northwest India presented with a longstanding headache that had worsened during the past 2 weeks and was associated with multiple episodes of vomiting and weakness on the left half of her body. Her past history did not suggest an immunocompromised state, comorbidities, or contact with tuberculosis. A general physical survey, including her nutritional status, was normal. Neurological examination revealed left hemiparesis and upper motor neuron facial paresis. Magnetic resonance imaging revealed a ring-enhancing lesion with perilesional edema in the basal ganglia and thalamic region (Figure 1C), along with a lipid peak on spectroscopy. The center of the lesion was markedly hypointense on T2-weighted imaging, suggesting caseous necrosis (Figure 1A, B). The typical appearance suggested a tuberculoma, which was confirmed with stereotactic biopsy.

**Figure 2.**
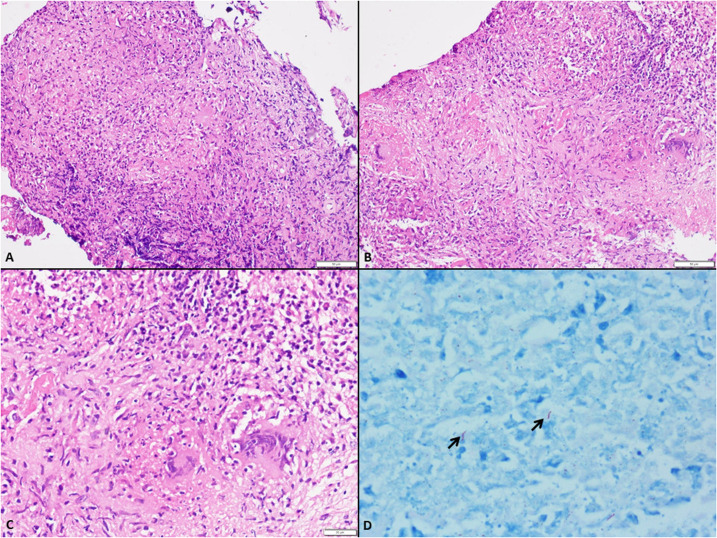
(**A**, **B**) Low magnification depicting loose clusters of epithelioid cells with giant cells forming granuloma; some are associated with central necrosis (hematoxylin–eosin stain; scale, 50 µm). (**C**) High magnification of epithelioid cell granuloma (hematoxylin–eosin stain; scale, 20 µm). (**D**) Ziehl–Neelsen stain highlights acid-fast bacilli (arrows) (×1,000 magnification). This figure appears in color at www.ajtmh.org.

Microscopy showed foci of necrosis surrounded by epithelioid histiocytes and dense lymphomononuclear cell infiltrate admixed with neutrophils and occasional giant cells; the specimen stained positive for acid-fast bacilli (Figure 2). A pulmonary workup for tuberculosis and serum HIV testing was unremarkable. The patient received anti-tuberculous therapy for 1 year with a combination of isoniazid (H), rifampicin (R), pyrazinamide (Z), and ethambutol (E) (3 months of HRZE, 9 months of HR) along with dexamethasone for the initial 6 weeks. Her symptoms had improved at the follow-up.

Central nervous system tuberculosis constitutes about 1% to 5% of all cases of tuberculosis.^1^ A diffuse involvement of the brain manifests as leptomeningitis, whereas localized disease gives rise to tuberculomas, abscesses, and cerebritis.^2^ Tuberculomas occur when the hematogenous spread of mycobacterial infection leads to formation of microgranulomatous foci (Rich foci), which coalesce to form non-caseating and caseating granulomas.^1^ These lesions contain epithelioid cells and Langhans giant cells with central caseation. The imaging depends on the pathological stage (i.e., caseating or non-caseating) of the center of the tuberculoma.^1^ A lesion with a caseating solid center, such as the one described here, depicts ring enhancement with a T2 hypointense appearance. The differentials to be borne in mind for a suspected case of tuberculoma include primary central nervous system lymphoma and other infectious etiologies such as fungal granulomas, toxoplasmosis, and cysticercosis.^1^

Cerebral tuberculoma is essentially a medical disease. A clinical–radiological suspicion combined with precise diagnosis obviates the unwarranted surgery-related morbidities especially in eloquent areas of the brain.
